# The Design and Synthesis of *N*-Xanthone Benzenesulfonamides as Novel Phosphoglycerate Mutase 1 (PGAM1) Inhibitors

**DOI:** 10.3390/molecules23061396

**Published:** 2018-06-08

**Authors:** Penghui Wang, Lulu Jiang, Yang Cao, Deyong Ye, Lu Zhou

**Affiliations:** Department of Medicinal Chemistry, School of Pharmacy, Fudan University, No. 826, Zhangheng Rd., Shanghai 201203, China; wangph@fudan.edu.cn (P.W.); lljiang16@fudan.edu.cn (L.J.); ycao14@fudan.edu.cn (Y.C.)

**Keywords:** phosphoglycerate mutase 1, inhibitors, anti-cancer, xanthone

## Abstract

Upregulation of phosphoglycerate mutase 1 (PGAM1) has been identified as one common phenomenon in a variety of cancers. Inhibition of PGAM1 provides a new promising therapeutic strategy for cancer treatment. Herein, based on our previous work, a series of new *N*-xanthone benzenesulfonamides were discovered as novel PGAM1 inhibitors. The representative molecule **15h**, with an IC_50_ of 2.1 μM, showed an enhanced PGAM1 inhibitory activity and higher enzyme inhibitory specificity compared to PGMI-004A, as well as a slightly improved antiproliferative activity.

## 1. Introduction

Metabolic reprogramming has been considered as one of 10 essential hallmarks of cancer cells [1]. This metabolic phenotype is associated with the phenomenon of cancer cells altering their metabolic pathways, including bioenergetics and anabolic biosynthesis, to satisfy the anabolic demands of macromolecular biosynthesis and to maintain cellular redox homeostasis in response to the escalated production of toxic reactive oxygen species (ROS) during cell proliferation [2]. The first identified cancer cell metabolism reprogramming phenomenon was the Warburg effect [3], which refers to cancer cells relying on the high rate of aerobic glycolysis to produce energy rather than the efficient mitochondrial oxidative phosphorylation as in most normal cells [4]. This specific metabolic pattern in cancer cells serves to supply glycolytic intermediates as building blocks for anabolic biosynthesis of macromolecules, such as RNA/DNA, proteins, and lipids [5]. More and more researchers have focused on the key enzymes in cancer cell metabolism reprogramming to find new cancer therapeutic targets [6,7,8,9,10].

Phosphoglycerate mutase 1 (PGAM1) is a glycolytic enzyme that catalyzes the interconversion of 3-phosphoglycerate (3PG) and 2-phosphoglycerate (2PG) with 2,3-bisphosphoglycerate (2,3-BPG) as a cofactor in glycolysis. PGAM1 was found to be upregulated in a variety of human cancers, including breast cancer [11], prostate cancer [12], lung cancer [13], etc. A recent work by Hitosugi showed that in cancer cells, upregulated PGAM1 coordinates glycolysis and biosynthesis to promote cancer cell proliferation and tumor growth [14]. The molecular mechanism of this function of PGAM1 is that the upregulation of PGAM1 leads to a lower intracellular level of 3PG and a higher intracellular level of 2PG, which results in a high level of pentose phosphate pathway (PPP) flux and activated serine synthesis pathway (SSP), respectively [14,15]. This mechanism facilitates the conversion of glycolytic intermediates to the precursors of amino acids and ribose, which are building blocks of DNA/RNA and proteins. In addition, both downregulation of PGAM1′s expression and inhibition of its metabolic activity has been shown to attenuate cell proliferation and tumor growth [14,16]. Accordingly, developing PGAM1 inhibitors could not only reduce the indispensable energy supply for cancer cells but also block the required anabolic processes including PPP and SSP, and hence provide a new dual-functional anticancer strategy [17].

**Figure 1:**
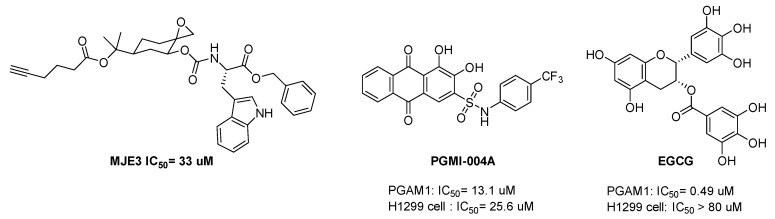
The inhibitors of PGAM1. Recently, starting from the PGMI-004A, we conducted scaffold hopping and a sulfonamide reversal strategy to discover a series of 1,2,8-trihydroxy xanthone derivatives as novel PGAM1 inhibitors (Figure 2). The inhibitory activities both on the enzymatic and cellular level of those xanthone derivatives were significantly improved compared to PGMI-004A [22]. Besides, the xanthone core as an important scaffold with diverse biologic activities, such as antitumor, antioxidant, anti-inflammation, etc., was of documented relevance to human diseases [23,24,25,26,27]. In this paper, maintaining the xanthone scaffold and considering that the *ortho*-dihydroxy phenol moiety might cause metabolic instability [28], we removed the C2-hydroxy group to design and synthesize a series of new *N*-xanthone benzenesulfonamides. What is more, the SAR of the A-ring has also been explored.

Although PGAM1 has been identified as one potential anticancer target, only three small molecules have currently been discovered as PGAM1 inhibitors (Figure 1). MJE3 was initially screened for inhibiting the proliferation of MDA-MB-231 cells and its antiproliferation effect was subsequently confirmed to come from its inhibitory activity against PGAM1 through in situ proteome reactivity profiling [18]. A further study showed that MJE3 could inactivate PGAM1 through covalent modification of PGAM1′s K100 by its spiroepoxide substructure [19]. PGMI-004A, with moderate enzymatic activity, showed a significant antiproliferation effect on both a cellular level and mice xenograft models [14]. Additionally, the third PGAM1 inhibitor, epigallocatechin gallate (EGCG), has recently been reported [20]. Although EGCG is the most potent PGAM1 inhibitor at the molecular level, its polyphenol structure and off-target effect may limit its further applications [21].

## 2. Results and Discussion

**Scheme 1:**
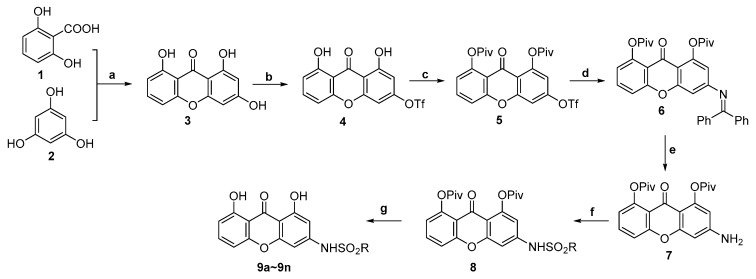
Reagents and conditions: (**a**) Eaton’s reagent (7.7 wt % P_2_O_5_ solution in MeSO_3_H), 80 °C; (**b**) Tf_2_O, pyridine, DCM, 0 °C; (**c**) NaH (60%), PivCl, THF; (**d**) Pd(OAc)_2_, BINAP, diphenylmethanimine, Cs_2_CO_3_, dioxane, reflux; (**e**) HCl, THF, H_2_O, r.t.; (**f**) RSO_2_Cl, pyridine, r.t.; (**g**) NaOH, MeOH, H_2_O, r.t.

First, to evaluate the substituent effects influencing the activities on the benzene ring, 14 *N*-xanthone benzenesulfonamides were synthesized as shown in Scheme 1. The xanthone core **3** was constructed by treating 2,6-dihydroxybenzoic acid **1** and phloroglucinol **2** with Eaton′s reagent and converted to triflate **4** by reacting with Tf_2_O. After pivaloylation of triflate **4**, the dipivalate **5** was obtained and was then converted to xanthone amine **7** through the Buchwald amination-hydrolysis protocol [29]. The xanthone amine **7** was treated with diverse substituted benzenesulfonyl chlorides and followed by deprotection to afford the target molecules *N*-xanthone benzenesulfonamides **9a**–**9n**.

A multiple-enzymes coupled assay system [14] was conducted to measure the inhibitory activities of the abovementioned compounds against PGAM1 (Table 1). The compounds with unsubstituted benzene (**9a** and **9b**) or benzene monosubstituted with halogens of low atomic weight (**9c**, **9d**, and **9e**) showed very low inhibitory activities against PGAM1 (inhibitory ratios at 20 uM were less than 50%), suggesting that the removal of the C2-hydroxy group would reduce the inhibitory activity. However, the iodo-substituted compound (**9f**) and the di-halogenated benzene compounds (**9g**, **9h**, and **9i**) restored the inhibitory activities, which indicating that bulky groups with enhanced lipophilicity might be beneficial for the inhibitory activity and counteract the decrease in activity due to the removal of the C2-hydroxyl group. Thus, large groups including (*tert*-butyl)phenyl (**9k**), cyclohexylphenyl (**9l**), biphenyl (**9m**), and naphthalenyl (**9n**) were introduced to the benzene ring of the benzenesulfonamides. As expected, the inhibitory activities of the compounds with large substituents is significantly increased compared to methyl substituted compounds **9j**. Among them, **9m** (IC_50_ = 5.5 ± 1.1 μM), containing a biphenyl moiety, was the most effective PGAM1 inhibitor with an IC_50_ value 2-fold lower than the reference inhibitor PGMI-004A. In general, the substituent effects of the benzene ring was consistent with our previous work.

**Scheme 2:**
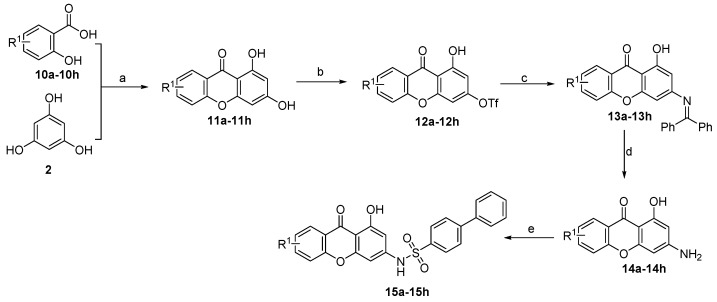
Reagents and conditions: (**a**) Eaton’s reagent (7.7 wt % P_2_O_5_ solution in MeSO_3_H), 80 °C; (**b**) Tf_2_O, pyridine, DCM, 0 °C; (**c**) Pd(OAc)_2_, BINAP, diphenylmethanimine, Cs_2_CO_3_, dioxane, reflux; (**d**) HCl, THF, H_2_O, r.t.; (**e**) [1,1′-biphenyl]-4-sulfonyl chloride, pyridine, r.t.

**Scheme 3:**
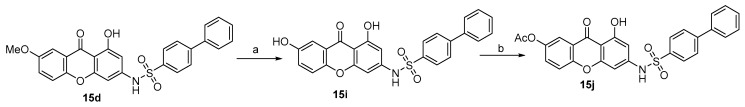
Reagents and conditions: (**a**) BBr_3_, DCM, 0 °C; (**b**) Ac_2_O, NEt_3_, DCM.

Further modification of **9m** was conducted at the A-ring of the xanthone core. Considering that the substituents of A-ring was derived from the starting material substituted salicylic acids, a series of A-ring substituted compounds were synthesized according to Scheme 2 and Scheme 3 and their enzymatic inhibitory activities were evaluated by the previous enzyme assay method (Table 2). Further analysis of the inhibitory activities gave us a preliminary structure–activity relationship (SAR) of the substituents in the A-ring. All the 5- or 7-substituted xanthones showed a better inhibitory activity than the unsubstituted compound **15a**, indicating that substitution on A-ring might increase the interaction of the inhibitors with PGAM1. Furthermore, the inhibitory effect of **15d** (7-OMe) and **15e** (7-Me) were slightly better than **15b** (5-OMe) and **15c** (5-Me), respectively. The 7-chloro substituted compounds **15f** with an IC_50_ of 3.5 μM could significantly enhance the activity, while its fluor-substituted homologs **15g** (7-F) was as active as the unsubstituted compounds **15a**. Comparing **15d** (IC_50_ = 4.6 μM) with **15e** (IC_50_ = 8.0 μM), we found that introducing the oxygen atom was beneficial for activity. We surmised that the oxygen atom might form hydrogen bonds with protein residues. Demethylation of **15d** (IC_50_ = 4.6 μM) afforded **15i** (IC_50_ = 6.4 μM), with an exposed hydroxyl group, slightly decreased the inhibitory activity compared to **15d.** For acetylation of the exposed hydroxyl group of **15i** to afford **15j**, the latter showed significantly enhanced inhibitory activity. In addition, the 7-NO_2_-substituted compound **15h**, with an IC_50_ of 2.1 μM, was the most potent inhibitor among the A-ring substituted xanthones.

In order to evaluate the specificity of these *N*-xanthone benzenesulfonamides against PGAM1, 13 compounds with IC_50_ less than 10 μM were selected to measure the inhibitory activities against 3 downstream enzymes. The counter-screen assay [14,22] was then performed to exclude the off-target inhibition against other enzymes in the in vitro assay (Table 3). All 13 *N*-xanthone benzenesulfonamides showed a much lower inhibitory effect on the counter-screen assay compared to PGMI-004A, which suggests that the *N*-xanthone benzenesulfonamides selectively inhibit PGAM1 in the multiple-enzymes coupled assay system.

**Figure 3:**
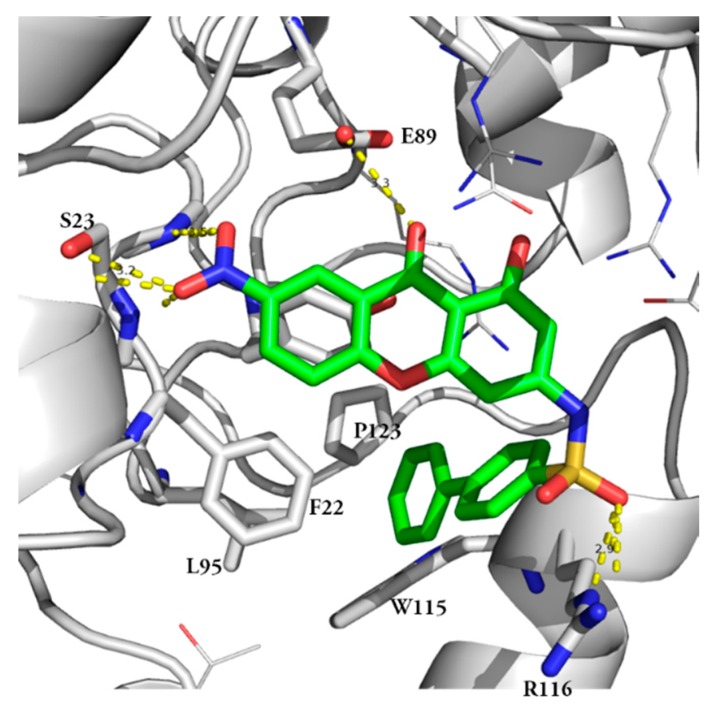
The docking model of **15h** with PGAM1.

To better understand the structure–activity relationship of the A-ring of the xanthone core, **15h** was docked into the crystal structure of PGAM1. The interactions between **15h** and PGAM1 are shown in Figure 3. The biphenyl group occupied the hydrophobic pocket formed by P123, F22, L95, and W115. The carbonyl and the sulfone amide group in the **15h** contacted with E89 and R116 through hydrogen bonds, respectively. In addition, the 7-nitro group formed two hydrogen bonds with S23, which enhanced the intramolecular interaction between **15h** and the enzyme.

After two rounds of optimization, the enzymatic inhibitory activity (IC_50_) against PGAM1 of these *N*-xanthone benzenesulfonamides was increased from 20 μM to 2.1 μM. Then, some representative compounds were picked out to evaluate their antiproliferative activity (H1299 cell line) by the MTT method (Table 4). Among them, **9h** showed a relatively strong inhibitory activity against H1299 cell proliferation.

## 3. Experimental Section

### 3.1. Chemistry Experimental Procedures and Compound Characterization

**1,3,8-Trihydroxy-9*H*-xanthen-9-one (3).** 2,6-dihydroxybenzoic acid (5.0 g, 32.4 mmol) and phloroglucinol (4.09 g, 32.4 mmol) were added to Eaton’s reagent (20 mL) and heated on an 80 °C oil bath for 2 h. After cooling to r.t., the dark-brown solution was transferred slowly to crushed ice while being vigorously stirred. The mixture was filtered and the filter cake was washed with water, dried, and purified by flash column chromatography to afford xanthone **3** as a yellow solid (2.3 g, yield: 29%). ^1^H NMR (400 MHz, DMSO-*d*_6_) δ 11.85 (s, 1H), 11.80 (s, 1H), 11.28 (s, 1H), 7.68 (tt, *J* = 8.3, 2.4 Hz, 1H), 7.00 (dt, *J* = 8.3, 3.3 Hz, 1H), 6.79 (ddd, *J* = 8.4, 3.0, 1.8 Hz, 1H), 6.39 (d, *J* = 1.9 Hz, 1H), 6.23 (d, *J* = 1.5 Hz, 1H).

**1,8-Dihydroxy-9-oxo-9*H*-xanthen-3-yl-trifluoromethanesulfonate (4).** To a solution of compound **3** (2.0 g, 8.2 mmol) and NEt_3_ (1.4 mL, 9.8 mmol) in dry DCM (20 mL), Tf_2_O (1.5 mL, 9.0 mmol) was added slowly at 0 °C. The reaction was completed in 2 h, shown by TLC analysis, and was then diluted with DCM (20 mL) and hydrochloric acid (1 mol/L, 40 mL). The organic phase was separated, washed with brine, dried over Na_2_SO_4_, filtered, and concentrated in vacuo successively. The residue was purified by flash column chromatography to afford triflate **4** as a yellow solid (1.5 g, yield: 48%). ^1^H NMR (400 MHz, DMSO) δ 12.15 (s, 1H), 11.58 (s, 1H), 7.78 (t, *J* = 8.4 Hz, 1H), 7.36 (d, *J* = 2.2 Hz, 1H), 7.08 (d, *J* = 8.4 Hz, 1H), 7.04 (d, *J* = 2.2 Hz, 1H), 6.88 (d, *J* = 8.3 Hz, 1H).

**9-Oxo-3-(((trifluoromethyl)sulfonyl)oxy)-9*H*-xanthene-1,8-diyl bis(2,2-dimethylpropanoate) (5).** To a solution of triflate **4** (1.0 g, 2.66 mmol) in dry THF (20 mL), 60% NaH (532 mg, 13.3 mmol) was added portionwise at 0 °C. After 20 min, pivaloyl chloride (0.98 mL, 8.0 mmol) was added slowly. The reaction was kept at 0 °C for another 1 h and then diluted with ethyl acetate (40 mL) and quenched with sat. NH_4_Cl solution (60 mL). The organic phase was separated, washed with brine, dried over Na_2_SO_4_, filtered, and concentrated in vacuo successively. The residue was recrystallized from methanol/ethyl acetate to afford the dipivalate **5** as a white solid (1.3 g, yield: 90%).

**3-((Diphenylmethylene)amino)-9-oxo-9*H*-xanthene-1,8-diyl bis(2,2-dimethylpropanoate) (6).** The dipivalate **5** (1.0 g, 1.84 mmol), benzophenone imine (666 mg, 3.67 mmol), BINAP (172 mg, 0.275 mmol), Pd(OAc)_2_ (41 mg, 0.184 mmol), and Cs_2_CO_3_ (898 mg, 2.75 mmol) were suspended in dioxane (30 mL) under argon and the resulting mixture was heated to reflux for 12 h. After cooling to r.t., the reaction mixture was diluted with ethyl acetate (50 mL), filtered through a celite bed and concentrated in vacuo successively. The residue was purified by flash column chromatography to afford the desired product **6** as a yellow solid (821 mg, yield: 77%).

**3-Amino-9-oxo-9*H*-xanthene-1,8-diyl bis(2,2-dimethylpropanoate) (7).** To a solution of compound **6** (168 mg, 0.372 mmol) in THF (10 mL), 4 *M* hydrochloric acid (4 mL) was added. After 30 min, the reaction mixture was diluted with ethyl acetate (10 mL) and quenched with saturated aqueous sodium bicarbonate solution (15 mL). The organic phase was separated, washed with brine, dried over sodium sulfate, and concentrated in vacuo successively. The residue was crystallized in hexane/ethyl acetate to afford the desired amine **7** as a yellow solid (85 mg, yield: 80%). ^1^H NMR (400 MHz, DMSO) δ 7.73–7.69 (m, 1H), 7.42 (dd, *J* = 8.5, 1.0 Hz, 1H), 6.95 (dd, *J* = 7.9, 1.0 Hz, 1H), 6.65 (brs, 2H), 6.41 (d, *J* = 2.1 Hz, 1H), 6.21 (d, *J* = 2.1 Hz, 1H), 1.35 (s, 9H), 1.33 (s, 9H).

**The general synthetic procedure of compounds 9a–9n.** To a solution of amine **7** (41 mg, 0.1 mmol) in dry pyridine (2 mL), substituted benzenesulfonyl chloride (1.5–2 eq.) was added. The reaction mixture was kept at r.t. overnight and poured into a mixture of 1 *M* hydrochloric acid (10 mL) and ethyl acetate (10 mL) while being vigorously stirred. The organic phase was separated and concentrated in vacuo. The residue was dissolved in a mixture of methanol (10 mL) and 5 *M* sodium hydroxide solution (5 mL) and kept at r.t. for 1 h. The mixture was concentrated in vacuo to remove the methanol and was diluted with water (3 mL) and filtered. The clear water phase was washed with ethyl acetate (3 mL × 2) and then concentrated hydrochloric acid was added dropwise until pH = 4. The mixture was filtered to afford the desired sulfonamide **9a**–**9n** as yellow solids (yield: 30–80%).

***N*-(1,8-Dihydroxy-9-oxo-9*H*-xanthen-3-yl)-1-phenylmethanesulfonamide (9a).** Yield: 53%. Yellow solid. M.p = 210–212 °C. R*_f_* = 0.35 (Petroleum ether: Acetone = 4:1). ^1^H NMR (400 MHz, DMSO-*d*_6_) δ 11.79 (s, 1H), 11.77 (s, 1H), 10.75 (s, 1H), 7.73 (t, *J* = 8.4 Hz, 1H), 7.39–7.33 (m, 3H), 7.32–7.26 (m, 2H), 7.07 (d, *J* = 8.4 Hz, 1H), 6.83 (d, *J* = 8.3 Hz, 1H), 6.78 (s, 1H), 6.56 (s, 1H), 4.71 (s, 2H). MS (ESI^−^) *m/z* 396.0 (M − H)^−^. HRMS (ESI^−^): Calcd. for C_20_H_14_NO_6_S^−^ [M − H]^−^ *m/z*: 396.0547, found: 396.0559.

***N*-(1,8-Dihydroxy-9-oxo-9*H*-xanthen-3-yl)benzenesulfonamide (9b).** Yield: 68%. Yellow solid. M.p = 260–263 °C. R*_f_* = 0.33 (Petroleum ether: Acetone = 4:1). ^1^H NMR (400 MHz, DMSO-*d*_6_) δ 11.71 (s, 1H), 11.69 (s, 1H), 11.40 (s, 1H), 7.92 (d, *J* = 7.9 Hz, 2H), 7.73–7.56 (m, 4H), 7.02 (d, *J* = 8.6 Hz, 1H), 6.79 (d, *J* = 8.3 Hz, 1H), 6.72 (s, 1H), 6.53 (s, 1H). ^13^C NMR (151 MHz, DMSO) δ 183.76, 161.25, 160.24, 156.69, 155.58, 146.64, 139.10, 137.64, 133.67, 129.74, 126.69, 110.81, 107.30, 107.26, 103.46, 99.41, 95.48. MS (ESI^−^) *m/z* 382.0 (M − H)^−^. HRMS (ESI^−^): Calcd. for C_19_H_12_NO_6_S^−^ [M − H]^−^ *m/z*: 382.0391, found: 382.0400.

***N*-(1,8-Dihydroxy-9-oxo-9*H*-xanthen-3-yl)-4-fluorobenzenesulfonamide (9c).** Yield: 64%. Yellow solid. M.p = 241–242 °C. R*_f_* = 0.30 (Petroleum ether: Acetone = 4:1). ^1^H NMR (400 MHz, DMSO-*d*_6_) δ 11.72 (s, 1H), 11.69 (s, 1H), 11.47 (s, 1H), 8.00 (dd, *J* = 8.7, 5.0 Hz, 2H), 7.69 (t, *J* = 8.3 Hz, 1H), 7.49 (t, *J* = 8.6 Hz, 2H), 7.02 (d, *J* = 8.4 Hz, 1H), 6.80 (d, *J* = 8.2 Hz, 1H), 6.73 (s, 1H), 6.54 (s, 1H). ^13^C NMR (151 MHz, DMSO) δ 183.80, 164.71 (d, *J* = 252.8 Hz), 161.28, 160.25, 156.71, 155.58, 146.35, 137.68, 135.40, 129.91 (d, *J* = 9.6 Hz), 117.02 (d, *J* = 22.9 Hz), 110.84, 107.32, 107.26, 103.61, 99.50, 95.59. MS (ESI^−^) *m/z* 399.9 (M − H)^−^. HRMS (ESI^−^): Calcd. for C_19_H_11_FNO_6_S^−^ [M − H]^−^ *m*/*z*: 400.0297, found: 400.0302.

***N*-(1,8-Dihydroxy-9-oxo-9*H*-xanthen-3-yl)-2-fluorobenzenesulfonamide (9d).** Yield: 75%. Yellow solid. M.p = 244–246 °C. R*_f_* = 0.30 (Petroleum ether: Acetone = 4:1). ^1^H NMR (400 MHz, DMSO-*d*_6_) δ 11.73 (s, 1H), 11.69 (s, 1H), 11.55 (s, 1H), 7.76–7.58 (m, 4H), 7.02 (d, *J* = 8.4 Hz, 1H), 6.80 (d, *J* = 8.3 Hz, 1H), 6.75 (s, 1H), 6.55 (s, 1H). ^13^C NMR (151 MHz, DMSO) δ 183.80, 161.27, 160.23, 158.20 (d, *J* = 255.2 Hz), 156.68, 155.59, 137.66, 136.79 (d, *J* = 7.8 Hz), 130.51, 126.51 (d, *J* = 13.2 Hz), 125.40, 117.68 (d, *J* = 20.1 Hz), 110.83, 107.33, 107.27, 103.54, 99.15, 95.27, 90.84. MS (ESI^−^) *m/z* 399.9 (M − H)^−^. HRMS (ESI^−^): Calcd. for C_19_H_11_FNO_6_S^−^ [M − H]^−^ *m/z*: 400.0297, found: 400.0307.

**4-Chloro-*N*-(1,8-dihydroxy-9-oxo-9*H*-xanthen-3-yl)benzenesulfonamide (9e).** Yield: 56%. Yellow solid. M.p = 257–258 °C. R*_f_* = 0.37 (Petroleum ether: Acetone = 4:1). ^1^H NMR (400 MHz, DMSO-*d*_6_) δ 11.72 (s, 1H), 11.68 (s, 1H), 11.47 (s, 1H), 7.92 (d, *J* = 8.2 Hz, 2H), 7.76–7.60 (m, 3H), 7.01 (d, *J* = 8.4 Hz, 1H), 6.79 (d, *J* = 8.4 Hz, 1H), 6.72 (s, 1H), 6.53 (s, 1H). ^13^C NMR (151 MHz, DMSO) δ 183.81, 161.30, 160.25, 156.72, 155.58, 146.23, 138.61, 137.87, 137.69, 129.93, 128.68, 110.85, 107.33, 107.26, 103.68, 99.56, 95.65. MS (ESI^−^) *m/z* 416.0 (M − H)^−^. HRMS (ESI^−^): Calcd. for C_19_H_11_ClNO_6_S^−^ [M − H]^−^ *m/z*: 416.0001, found: 416.0011.

***N*-(1,8-Dihydroxy-9-oxo-9*H*-xanthen-3-yl)-4-iodobenzenesulfonamide (9f).** Yield: 69%. Yellow solid. M.p = 269–272 °C. R*_f_* = 0.32 (Petroleum ether: Acetone = 4:1). ^1^H NMR (400 MHz, DMSO-*d*_6_) δ 11.79 (s, 1H), 11.69 (s, 1H), 7.99 (d, *J* = 8.1 Hz, 2H), 7.76–7.56 (m, 3H), 7.00 (d, *J* = 8.1 Hz, 1H), 6.78 (d, *J* = 8.1 Hz, 1H), 6.64 (s, 1H), 6.44 (s, 1H). ^13^C NMR (151 MHz, DMSO) δ 183.34, 161.13, 160.23, 156.71, 155.55, 138.78, 138.37, 137.39, 128.97, 128.25, 110.68, 107.20, 102.77, 101.28, 100.05, 95.92. MS (ESI^−^) *m/z* 508.0 (M − H)^−^. HRMS (ESI^−^): Calcd. for C_19_H_11_INO_6_S^−^ [M − H]^−^ *m/z*: 507.9357, found: 507.9376.

***N*-(1,8-Dihydroxy-9-oxo-9*H*-xanthen-3-yl)-3,5-difluorobenzenesulfonamide (9g).** Yield: 80%. Yellow solid. M.p = 272–274 °C. R*_f_* = 0.34 (Petroleum ether: Acetone = 4:1). ^1^H NMR (400 MHz, DMSO-*d*_6_) δ 11.73 (s, 1H), 11.69 (s, 1H), 11.55 (s, 1H), 7.78–7.54 (m, 4H), 7.02 (d, *J* = 8.4 Hz, 1H), 6.80 (d, *J* = 8.3 Hz, 1H), 6.75 (s, 1H), 6.55 (s, 1H). ^13^C NMR (151 MHz, DMSO) δ 183.73, 163.04 (d, *J* = 12.6 Hz), 161.37 (d, *J* = 12.3 Hz), 161.20, 160.16, 156.65, 155.50, 145.80, 142.25, 137.59, 110.88–110.30 (m), 109.58 (t, *J* = 25.6 Hz), 107.28, 107.16, 103.82, 99.76, 95.93. 161.41. MS (ESI^−^) *m/z* 417.8 (M − H)^−^. HRMS (ESI^−^): Calcd. for C_19_H_10_F_2_NO_6_S^−^ [M − H]^−^ *m/z*: 418.0202, found: 418.0212.

**3-Chloro-*N*-(1,8-dihydroxy-9-oxo-9*H*-xanthen-3-yl)-2-fluorobenzenesulfonamide (9h).** Yield: 64%. Yellow solid. M.p = 225–227 °C. R*_f_* = 0.31 (Petroleum ether: Acetone = 4:1). ^1^H NMR (400 MHz, DMSO-*d*_6_) δ 11.89 (s, 1H), 11.71 (s, 2H), 7.98 (dt, *J* = 22.0, 7.4 Hz, 2H), 7.68 (t, *J* = 8.4 Hz, 1H), 7.49 (t, *J* = 8.1 Hz, 1H), 7.00 (d, *J* = 8.4 Hz, 1H), 6.79 (d, *J* = 8.3 Hz, 1H), 6.68 (s, 1H), 6.51 (s, 1H). ^13^C NMR (151 MHz, DMSO) δ 183.64, 161.81, 161.26, 160.24, 156.72, 155.58, 153.61 (d, *J* = 256.5 Hz), 137.57, 136.37, 129.25, 126.13, 121.68 (d, *J* = 16.7 Hz), 110.78, 107.30, 107.24, 99.52, 95.62, 95.35, 90.84. MS (ESI^−^) *m/z* 434.0 (M − H)^−^. HRMS (ESI^−^): Calcd. for C_19_H_14_ClFNO_6_S^−^ [M − H]^−^ *m/z*: 433.9907, found: 433.9917.

**2,6-Dichloro-*N*-(1,8-dihydroxy-9-oxo-9*H*-xanthen-3-yl)benzenesulfonamide (9i).** Yield: 43%. Yellow solid. M.p = 238–239 °C. R*_f_* = 0.37 (Petroleum ether: Acetone = 4:1). ^1^H NMR (400 MHz, DMSO-*d*_6_) δ 11.89 (s, 1H), 11.75 (s, 1H), 11.65 (s, 1H), 7.78–7.53 (m, 4H), 7.02 (d, *J* = 8.4 Hz, 1H), 6.80 (d, *J* = 8.1 Hz, 1H), 6.68 (s, 1H), 6.52 (s, 1H). ^13^C NMR (151 MHz, DMSO) δ 183.74, 161.26, 160.11, 156.60, 155.49, 145.56, 137.56, 134.78, 134.41, 133.07, 132.15, 110.75, 107.23, 107.19, 103.44, 98.61, 94.86. MS (ESI^−^) *m/z* 450.0 (M−H)^−^. HRMS (ESI^−^): Calcd. for C_19_H_10_Cl_2_NO_6_S^−^ [M−H]^−^ *m/z*: 449.9611, found: 449.9621.

***N*-(1,8-Dihydroxy-9-oxo-9*H*-xanthen-3-yl)-4-methylbenzenesulfonamide (9j).** Yield: 80%. Yellow solid. M.p = 218–220 °C. R*_f_* = 0.35 (Petroleum ether: Acetone = 4:1). ^1^H NMR (400 MHz, DMSO) δ 11.71 (s, 2H), 11.33 (brs, 1H), 7.80 (d, *J* = 8.4 Hz, 2H), 7.70 (t, *J* = 8.4 Hz, 1H), 7.43 (d, *J* = 8.0 Hz, 2H), 7.03 (d, *J* = 8.4 Hz, 1H), 6.80 (d, *J* = 8.3 Hz, 1H), 6.71 (d, *J* = 1.9 Hz, 1H), 6.52 (d, *J* = 1.9 Hz, 1H), 2.35 (s, 3H). ^13^C NMR (151 MHz, DMSO) δ 183.44, 161.00, 160.06, 156.48, 155.39, 147.33, 143.78, 137.36, 136.41, 129.88, 126.55, 110.58, 107.07, 107.04, 102.97, 99.33, 95.34, 20.82. MS (ESI^−^) *m/z* 396.0 (M − H)^−^. HRMS (ESI^−^): Calcd. for C_20_H_14_NO_6_S^−^ [M − H]^−^ *m/z*: 396.0547, found: 396.0553.

**4-(*tert*-Butyl)-*N*-(1,8-dihydroxy-9-oxo-9*H*-xanthen-3-yl)benzenesulfonamide (9k).** Yield: 67%. Yellow solid. M.p = 205–208 °C. R*_f_* = 0.43 (Petroleum ether: Acetone = 4:1). ^1^H NMR (400 MHz, DMSO-*d*_6_) δ 11.73 (s, 1H), 11.70 (s, 1H), 11.38 (s, 1H), 7.84 (d, *J* = 8.2 Hz, 2H), 7.73–7.61 (m, 3H), 7.01 (d, *J* = 8.5 Hz, 1H), 6.79 (d, *J* = 8.3 Hz, 1H), 6.72 (s, 1H), 6.52 (s, 1H), 1.26 (s, 9H). ^13^C NMR (151 MHz, DMSO) δ 183.40, 161.08, 160.13, 156.62, 155.46, 137.36, 136.24, 126.39, 126.36, 110.62, 110.22, 107.12, 106.76, 99.41, 95.35, 95.24, 90.73, 34.83, 30.60. MS (ESI^−^) *m/z* 438.0 (M − H)^−^. HRMS (ESI^−^): Calcd. for C_23_H_20_NO_6_S^−^ [M − H]^−^ *m/z*: 438.1017, found: 438.1030.

**4-Cyclohexyl-*N*-(1,8-dihydroxy-9-oxo-9*H*-xanthen-3-yl)benzenesulfonamide (9l).** Yield: 66%. Yellow solid. M.p = 203-205 °C. R*_f_* = 0.51 (Petroleum ether: Acetone = 4:1). ^1^H NMR (400 MHz, DMSO-*d*_6_) δ 11.71 (s, 1H), 11.69 (s, 1H), 11.36 (s, 1H), 7.83 (d, *J* = 8.1 Hz, 2H), 7.68 (t, *J* = 8.4 Hz, 1H), 7.49 (d, *J* = 8.0 Hz, 2H), 7.01 (d, *J* = 8.4 Hz, 1H), 6.79 (d, *J* = 8.3 Hz, 1H), 6.74 (s, 1H), 6.55 (s, 1H), 2.63–2.51 (m, 1H), 1.79–1.60 (m, 5H), 1.44–1.12 (m, 5H). ^13^C NMR (151 MHz, DMSO) δ 183.65, 161.15, 160.14, 156.61, 155.47, 153.47, 146.56, 137.52, 136.53, 127.89, 126.72, 110.70, 107.19, 107.15, 103.30, 99.13, 95.19, 43.42, 33.25, 25.99, 25.29. MS (ESI^−^) *m/z* 464.1 (M − H)^−^. HRMS (ESI^−^): Calcd. for C_25_H_22_NO_6_S^−^ [M − H]^−^ *m/z*: 464.1173, found: 464.1187.

***N*-(1,8-Dihydroxy-9-oxo-9*H*-xanthen-3-yl)-[1,1′-biphenyl]-4-sulfonamide (9m).** Yield: 74%. Yellow solid. M.p = 279–280 °C. R*_f_* = 0.55 (Petroleum ether: Acetone = 4:1). ^1^H NMR (400 MHz, DMSO-*d*_6_) δ 11.71 (s, 1H), 11.67 (s, 1H), 11.49 (s, 1H), 8.00 (d, *J* = 8.3 Hz, 2H), 7.93 (d, *J* = 8.3 Hz, 2H), 7.74–7.62 (m, 3H), 7.51–7.39 (m, 3H), 7.00 (d, *J* = 8.3 Hz, 1H), 6.77 (d, *J* = 6.8 Hz, 2H), 6.59 (s, 1H). ^13^C NMR (151 MHz, DMSO) δ 183.59, 161.11, 160.06, 156.54, 155.39, 146.38, 144.86, 137.93, 137.63, 137.45, 128.96, 128.56, 127.68, 127.23, 126.94, 110.63, 107.11, 107.06, 103.34, 99.24, 95.30. MS (ESI^−^) *m/z* 458.0 (M − H)^−^. HRMS (ESI^−^): Calcd. for C_25_H_16_NO_6_S^−^ [M − H]^−^ *m/z*: 458.0704, found: 458.0718.

***N*-(1,8-Dihydroxy-9-oxo-9*H*-xanthen-3-yl)naphthalene-1-sulfonamide (9n).** Yield: 76%. Yellow solid. M.p = 220–222 °C. R*_f_* = 0.52 (Petroleum ether: Acetone = 4:1). ^1^H NMR (400 MHz, DMSO-*d*_6_) δ 11.69 (s, 1H), 11.67 (s, 1H), 11.49 (s, 1H), 8.66 (s, 1H), 8.23 (d, *J* = 7.9 Hz, 1H), 8.15 (d, *J* = 8.8 Hz, 1H), 8.02 (d, *J* = 7.9 Hz, 1H), 7.86 (dd, *J* = 8.7, 1.9 Hz, 1H), 7.78–7.58 (m, 3H), 6.98 (d, *J* = 8.5 Hz, 1H), 6.82–6.71 (m, 2H), 6.56 (s, 1H). ^13^C NMR (151 MHz, DMSO) δ 183.45, 161.02, 160.04, 156.50, 155.38, 147.09, 137.35, 136.20, 134.28, 131.42, 129.77, 129.2-24, 129.13, 128.01, 127.73, 121.56, 110.57, 107.08, 107.04, 103.07, 99.39, 95.44. MS (ESI^−^) *m/z* 432.1 (M − H)^−^. HRMS (ESI^−^): Calcd. for C_23_H_14_NO_6_S^−^ [M − H]^−^ *m/z*: 432.0547, found: 432.0558.

**The general synthetic procedures of compounds 15a**–**15f**. The synthetic procedures of **15a**–**15f** were similar to **9n**.

***N*-(1-Hydroxy-9-oxo-9*H*-xanthen-3-yl)-[1,1′-biphenyl]-4-sulfonamide (15a).** Yield: 60%. Yellow solid. M.p = 234–235 °C. R*_f_* = 0.56 (Petroleum ether: Acetone = 4:1). ^1^H NMR (400 MHz, DMSO-*d*_6_) δ 12.67 (s, 1H), 11.40 (s, 1H), 8.10 (d, *J* = 7.6 Hz, 1H), 8.00 (d, *J* = 8.2 Hz, 2H), 7.93 (d, *J* = 8.1 Hz, 2H), 7.87 (t, *J* = 7.8 Hz, 1H), 7.71 (d, *J* = 7.8 Hz, 2H), 7.63 (d, *J* = 8.5 Hz, 1H), 7.54 – 7.36 (m, 4H), 6.81 (d, *J* = 1.8 Hz, 1H), 6.58 (d, *J* = 1.9 Hz, 1H). ^13^C NMR (151 MHz, DMSO) δ 180.18, 161.88, 156.55, 155.41, 145.99, 144.88, 138.02, 137.76, 136.02, 129.03, 128.62, 127.75, 127.28, 127.02, 125.16, 124.57, 119.80, 117.81, 104.30, 99.02, 95.35. MS (ESI^−^) *m/z* 442.1 (M − H)^−^. HRMS (ESI^−^): Calcd. for C_25_H_16_NO_5_S^−^ [M − H]^−^ *m/z*: 442.0705, found: 442.0716.

***N*-(1-Hydroxy-5-methoxy-9-oxo-9*H*-xanthen-3-yl)-[1,1′-biphenyl]-4-sulfonamide (15b).** Yield: 47%. Yellow solid. M.p = 220–221 °C. R*_f_* = 0.36 (Petroleum ether: Acetone = 4:1). ^1^H NMR (400 MHz, DMSO-*d*_6_) δ 12.66 (s, 1H), 11.42 (s, 1H), 8.01–7.89 (m, 5H), 7.72 (d, *J* = 7.7 Hz, 2H), 7.50 (q, *J* = 8.1, 7.5 Hz, 4H), 7.45–7.34 (m, 1H), 6.80 (s, 1H), 6.58 (s, 1H), 3.97 (s, 3H). ^13^C NMR (151 MHz, DMSO) δ 180.21, 161.79, 156.30, 147.88, 145.89, 145.44, 144.87, 138.00, 137.74, 129.02, 128.62, 127.77, 127.21, 127.02, 124.27, 120.56, 116.96, 115.58, 104.28, 99.13, 95.46, 56.21. MS (ESI^−^) *m/z* 471.8 (M − H)^−^. HRMS (ESI^−^): Calcd. for C_26_H_18_NO_6_S^−^ [M − H]^−^ *m/z*: 472.0860, found: 472.0878.

***N*-(1-Hydroxy-5-methyl-9-oxo-9*H*-xanthen-3-yl)-[1,1′-biphenyl]-4-sulfonamide (15c).** Yield: 48%. Yellow solid. M.p = 235–237 °C. R*_f_* = 0.50 (Petroleum ether: Acetone = 4:1). ^1^H NMR (400 MHz, DMSO-*d*_6_) δ 12.72 (s, 1H), 11.38 (s, 1H), 8.00 (d, *J* = 8.4 Hz, 2H), 7.96–7.88 (m, 3H), 7.72 (t, *J* = 7.9 Hz, 3H), 7.49 (t, *J* = 7.1 Hz, 2H), 7.43 (d, *J* = 6.6 Hz, 1H), 7.36 (t, *J* = 7.7 Hz, 1H), 6.83 (s, 1H), 6.59 (s, 1H), 2.48 (s, 3H). ^13^C NMR (151 MHz, DMSO) δ 180.38, 161.75, 156.36, 153.68, 145.88, 144.79, 137.95, 137.74, 136.61, 128.97, 128.56, 127.67, 127.19, 126.95, 126.74, 123.93, 122.68, 119.55, 104.05, 99.03, 95.57, 15.06. MS (ESI^−^) *m/z* 456.1 (M − H)^−^. HRMS (ESI^−^): Calcd. for C_26_H_18_NO_5_S^−^ [M − H]^−^ *m/z*: 456.0911, found: 456.0926.

***N*-(1-Hydroxy-7-methoxy-9-oxo-9*H*-xanthen-3-yl)-[1,1′-biphenyl]-4-sulfonamide (15d).** Yield: 58%. Yellow solid. M.p = 220–222 °C. R*_f_* = 0.33 (Petroleum ether: Acetone = 4:1). ^1^H NMR (400 MHz, DMSO-*d*_6_) δ 12.62 (s, 1H), 11.28 (s, 1H), 7.95–7.77 (m, 4H), 7.70–7.55 (m, 2H), 7.45–7.26 (m, 6H), 6.71 (s, 1H), 6.48 (s, 1H), 3.77 (s, 3H). ^13^C NMR (151 MHz, DMSO) δ 179.82, 161.68, 156.45, 155.70, 150.07, 145.72, 144.81, 137.95, 137.70, 128.96, 128.56, 127.68, 127.20, 126.95, 124.93, 120.11, 119.34, 105.03, 103.91, 98.80, 95.13, 55.63. MS (ESI^−^) *m/z* 471.9 (M − H)^−^. HRMS (ESI^−^): Calcd. for C_26_H_18_NO_6_S^−^ [M − H]^−^ *m/z*: 472.0860, found: 472.0873.

***N*-(1-Hydroxy-7-methyl-9-oxo-9*H*-xanthen-3-yl)-[1,1′-biphenyl]-4-sulfonamide (15e).** Yield: 60%. Yellow solid. M.p = 239–240. R*_f_* = 0.48 (Petroleum ether: Acetone = 4:1). ^1^H NMR (400 MHz, DMSO-*d*_6_) δ 12.72 (s, 1H), 11.39 (s, 1H), 7.99 (d, *J* = 8.4 Hz, 2H), 7.93 (d, *J* = 8.5 Hz, 2H), 7.87 (s, 1H), 7.71 (d, *J* = 7.2 Hz, 2H), 7.66 (d, *J* = 8.7 Hz, 1H), 7.56–7.33 (m, 4H), 6.79 (d, *J* = 1.8 Hz, 1H), 6.56 (d, *J* = 1.7 Hz, 1H), 2.40 (s, 3H). ^13^C NMR (151 MHz, DMSO) δ 180.10, 161.81, 156.47, 153.59, 145.80, 144.80, 137.95, 137.71, 137.01, 134.02, 128.96, 128.55, 127.68, 127.21, 126.95, 124.29, 119.36, 117.55, 104.20, 98.84, 95.22, 20.12. MS (ESI^−^) *m/z* 456.1 (M − H)^−^. HRMS (ESI^−^): Calcd. for C_26_H_18_NO_5_S^−^ [M − H]^−^ *m/z*: 456.0911, found: 456.0928.

***N*-(7-Chloro-1-hydroxy-9-oxo-9*H*-xanthen-3-yl)-[1,1′-biphenyl]-4-sulfonamide (15f).** Yield: 58%. Yellow solid. M.p = 259–260 °C. R*_f_* = 0.38 (Petroleum ether: Acetone = 4:1). ^1^H NMR (400 MHz, DMSO-*d*_6_) δ 12.42 (s, 1H), 11.45 (s, 1H), 8.05–7.95 (m, 3H), 7.96–7.86 (m, 3H), 7.75–7.66 (m, 3H), 7.49 (t, *J* = 7.3 Hz, 2H), 7.43 (d, *J* = 7.4 Hz, 1H), 6.81 (d, *J* = 2.0 Hz, 1H), 6.59 (d, *J* = 2.0 Hz, 1H). MS (ESI^−^) *m/z* 476.0 (M − H)^−^. HRMS (ESI^−^): Calcd. for C_25_H_15_ClNO_5_S^−^ [M − H]^−^ *m/z*: 476.0365, found: 476.0379.

***N*-(7-Fluoro-1-hydroxy-9-oxo-9*H*-xanthen-3-yl)-[1,1′-biphenyl]-4-sulfonamide (15g).** Yield: 51%. Yellow solid. M.p = 250–252 °C. R*_f_* = 0.45 (Petroleum ether: Acetone = 4:1). ^1^H NMR (400 MHz, DMSO-*d*_6_) δ 12.46 (s, 1H), 11.45 (s, 1H), 7.99 (d, *J* = 8.4 Hz, 2H), 7.93 (d, *J* = 8.8 Hz, 2H), 7.82–7.68 (m, 5H), 7.56–7.36 (m, 3H), 6.81 (s, 1H), 6.58 (s, 1H). ^13^C NMR (151 MHz, DMSO) δ 179.36, 161.61, 158.10 (d, *J* = 243.9 Hz), 156.53, 151.81, 146.17, 144.83, 137.93, 137.67, 128.97, 128.57, 127.69, 127.21, 126.96, 123.81 (d, *J* = 24.7 Hz), 120.76 (d, *J* = 7.4 Hz), 120.40 (d, *J* = 7.9 Hz), 109.78 (d, *J* = 24.3 Hz), 103.86, 99.07, 95.22. MS (ESI^−^) *m/z* 460.1 (M − H)^−^. HRMS (ESI^−^): Calcd. for C_25_H_15_FNO_5_S^−^ [M − H]^−^ *m/z*: 460.0660, found: 460.0677.

***N*-(1-Hydroxy-7-nitro-9-oxo-9*H*-xanthen-3-yl)-[1,1′-biphenyl]-4-sulfonamide (15h).** Yield: 35%. Yellow solid. M.p = 243-245. R*_f_* = 0.25 (Petroleum ether: Acetone = 4:1). ^1^H NMR (400 MHz, DMSO-*d*_6_) δ 12.21 (s, 1H), 11.51 (s, 1H), 8.76 (s, 1H), 8.64–8.55 (m, 1H), 8.00 (d, *J* = 8.1 Hz, 2H), 7.93 (d, *J* = 8.3 Hz, 2H), 7.86 (d, *J* = 9.6 Hz, 1H), 7.71 (d, *J* = 7.7 Hz, 2H), 7.48 (t, *J* = 7.6 Hz, 2H), 7.47–7.38 (m, 1H), 6.85 (s, 1H), 6.64 (s, 1H). ^13^C NMR (151 MHz, DMSO) δ 178.84, 161.67, 158.60, 156.39, 146.65, 144.95, 143.43, 137.97, 137.65, 129.79, 129.03, 128.64, 127.77, 127.28, 127.01, 121.11, 120.15, 119.90, 104.27, 99.76, 95.72. MS (ESI^−^) *m/z* 487.1 (M − H)^−^. HRMS (ESI^−^): Calcd. for C_25_H_15_N_2_O_7_S^−^ [M − H]^−^ *m/z*: 487.0605, found: 487.0626.

***N*-(1,7-Dihydroxy-9-oxo-9*H*-xanthen-3-yl)-[1,1′-biphenyl]-4-sulfonamide (15i).** To a solution of Compound **15d** (30 mg, 0.063 mmol) in dry DCM (5 mL), BBr_3_ (1 mol/L in DCM, 0.5 mL) was added. The reaction was kept at 0 °C for 2 h and then quenched with methanol (10 mL). The mixed solution was concentrated in vacuo and the residue was purified by flash column chromatography to give the demethylated product **15i** as a yellow solid (18 mg, yield: 61%). Yellow solid. M.p = 257–259 °C. R*_f_* = 0.53 (Petroleum ether: Acetone = 2:1). ^1^H NMR (400 MHz, DMSO-*d*_6_) δ 12.74 (s, 1H), 11.34 (s, 1H), 10.05 (s, 1H), 7.98 (d, *J* = 9.5 Hz, 2H), 7.92 (d, *J* = 9.3 Hz, 2H), 7.71 (d, *J* = 8.1 Hz, 2H), 7.56–7.13 (m, 6H), 6.77 (s, 1H), 6.54 (s, 1H). ^13^C NMR (151 MHz, DMSO) δ 179.99, 161.71, 156.49, 153.97, 148.98, 145.60, 144.79, 137.96, 137.71, 128.96, 128.55, 127.67, 127.19, 126.96, 124.87, 120.28, 119.06, 107.69, 103.86, 98.65, 95.08. MS (ESI^−^) *m/z* 458.1 (M − H)^−^. HRMS (ESI^−^): Calcd. for C_25_H_16_NO_6_S^−^ [M − H]^−^ *m/z*: 458.0704, found: 458.0710.

**6-([1,1′-Biphenyl]-4-sulfonamido)-8-hydroxy-9-oxo-9*H*-xanthen-2-yl acetate (15j).** To a solution of Compound **15i** (19 mg, 0.041 mmol) and NEt_3_ (0.017 mL, 0.124 mmol) in dry DCM (5 mL), Ac_2_O (0.006 mL, 0.062 mmol) was added. After the reaction was complete, which was monitored by TLC, the reaction mixture was concentrated in vacuo and the residue was purified by flash column chromatography to give the acetylated product **15j** as a yellow solid (16 mg, yield: 77%). Yellow solid. M.p = 261–262 °C. R*_f_* = 0.51 (Petroleum ether: Acetone = 4:1). ^1^H NMR (400 MHz, DMSO-*d*_6_) δ 12.52 (s, 1H), 11.45 (s, 1H), 8.00 (d, *J* = 8.2 Hz, 2H), 7.93 (d, *J* = 8.3 Hz, 2H), 7.82 (s, 1H), 7.77–7.61 (m, 4H), 7.53–7.37 (m, 3H), 6.81 (s, 1H), 6.58 (s, 1H), 2.30 (s, 3H). ^13^C NMR (151 MHz, DMSO) δ 179.65, 169.13, 161.74, 156.59, 152.90, 146.54, 146.06, 144.90, 138.00, 137.70, 130.13, 129.03, 128.63, 127.77, 127.27, 127.02, 120.30, 119.31, 117.22, 104.04, 99.05, 95.30, 20.73. MS (ESI^−^) *m/z* 500.0 (M − H)^−^. HRMS (ESI^−^): Calcd. for C_27_H_18_NO_7_S^−^ [M − H]^−^ *m/z*: 500.0809, found: 500.0831.

### 3.2. Biological Evaluation Assay

#### 3.2.1. In Vitro PGAM1 Enzyme Inhibitory Activity Assay

First, 1 µL of test compound (dissolved in DMSO) with a specific concentration, 1 µL of recombinant PGAM1 and 48 µL of Tris buffer solution (50 mM, pH 8.0), was added to a 96-well plate followed by preincubation for 2 min at room temperature. Then, 49 µL of enzyme buffer (containing 0.5 U/mL enolase, 0.5 U/mL PK, 0.1 U/mL LDH, 5 mM MgCl_2_, 1 mM ADP, 100 mM KCl, 0.2 mM NADH, 100 mM Tris pH 8.0) was added to the mixture. Finally, 1 µL of 3PG solution (200 mM) was added to initiate the reaction. The decrease in OD (λ = 340 nm) from the oxidation of NADH was measured by a microplate reader as PGAM1 activity.

#### 3.2.2. Counter-Screen Assay Activities of the Selected Compounds

First, 1 µL of test compound (dissolved in DMSO) with a specific concentration and 49 µL of Tris buffer solution (50 mM, pH 8.0) were added to a 96-well plate followed by preincubation for 2 min at room temperature. Then, 49 µL of enzymes buffer (containing 0.5 U/mL enolase, 0.5 U/mL PK, 0.1 U/mL LDH, 5 mM MgCl_2_, 1 mM ADP, 100 mM KCl, 0.2 mM NADH, 100 mM Tris pH 8.0) was added to the mixture. Finally, 1 µL of 2PG solution (200 mM) was added to initiate the reaction. The decrease in OD (λ = 340 nm) from the oxidation of NADH was measured as counter-screen assay activities by a microplate reader.

#### 3.2.3. In Vitro Antiproliferation of H1299 Cell Activity Assay

2 × 10^3^ cells were seeded in a 96-well plate before starting the assay and they were cultured at 37 °C. After seeding for 24 h, cells were treated with inhibitors with a specific concentration and incubated at 37 °C for 72 h, followed by incubation with 0.5 mg mL^−1^ MTT for 4 h at 37 °C. Then, 200 µL of DMSO was added and the absorbance was measured at 570 nm.

## 4. Conclusions

In summary, based on our previous work, we continued two rounds of modification to discover new *N*-xanthone benzenesulfonamides as PGAM1 inhibitors. A total number of 24 *N*-xanthone benzenesulfonamides were designed and synthesized, and their inhibitory activities against PGAM1 were evaluated. Among them, the most active and specific compound **15h** (IC_50_ = 2.1 μM) showed a 5-fold enhancement of PGAM1 inhibitory activity and a much higher specificity compared to PGMI-004A. Further, the antiproliferation activities on the H1299 cell line of the representative *N*-xanthone benzenesulfonamides were also evaluated, which showed a slightly increased antiproliferative activity. Consequently, in this study, we have expanded the structural types of PGAM1 inhibitors and provided a new direction for further development of more efficient PGAM1 inhibitors.

## Figures and Tables

**Figure 2:**
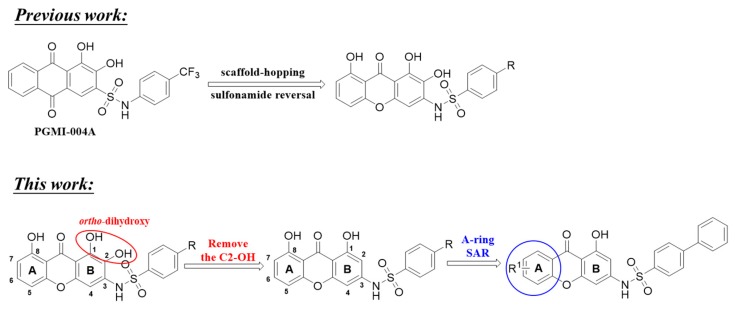
The optimization process of PGMI-004A.

**Table 1:**
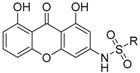
Inhibitory activities of **9a**–**9n** against PGAM1.

Entry	Compounds	R	IC_50_ (μM) *
1	**9a**	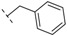	>20 **
2	**9b**	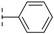	>20
3	**9c**	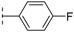	>20
4	**9d**	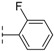	>20
5	**9e**	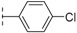	>20
6	**9f**	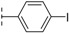	10.1 ± 0.5
7	**9g**	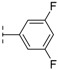	13.2 ± 0.4
8	**9h**	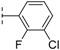	6.4 ± 0.4
9	**9i**	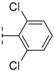	10.2 ± 0.7
10	**9j**	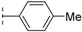	>20
11	**9k**	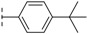	8.4 ± 2.7
12	**9l**	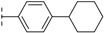	5.9 ± 1.2
13	**9m**	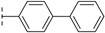	5.5 ± 1.1
14	**9n**	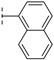	6.0 ± 0.6
15	PGMI-004A		13 ± 0.1

* All IC_50_ values are reported as means ± SD from three determinations. ** The inhibition ratios at 20 μM were less than 50%.

**Table 2:**
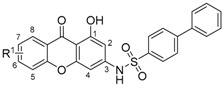
Inhibitory activities of **15a**–**15k** against PGAM1.

Entry	Compounds	R^1^	IC_50_ (μM) *
1	**9m**	8-OH	5.5 ± 1.1
2	**15a**	H	14.3 ± 0.8
3	**15b**	5-OMe	6.5 ± 1.3
4	**15c**	5-Me	8.6 ± 3.1
5	**15d**	7-OMe	4.6 ± 0.8
6	**15e**	7-Me	8.0 ± 2.1
7	**15f**	7-Cl	3.5 ± 1.0
8	**15g**	7-F	13.7 ± 5.5
9	**15h**	7-NO_2_	2.1 ± 0.2
10	**15i**	7-OH	6.4 ± 1.0
11	**15j**	7-OAc	2.7 ± 0.5
12	PGMI-004A		13 ± 0.1

* All IC_50_ values are reported as means ± SD from three determinations.

**Table 3:** The counter-screen assay of the representative compounds.

Entry	Compounds	PGAM1 IC_50_ (μM)	Counter-Screen Assay (5 μM) *
1	**9h**	6.4 ± 0.4	12%
2	**9k**	8.4 ± 2.7	12%
3	**9l**	5.9 ± 1.2	15%
4	**9m**	5.5 ± 1.1	9%
5	**9n**	6.0 ± 0.6	13%
6	**15b**	6.5 ± 1.3	13%
7	**15c**	8.6 ± 3.1	7%
8	**15d**	4.6 ± 0.8	6%
9	**15e**	8.0 ± 2.1	7%
10	**15f**	3.5 ± 1.0	8%
11	**15h**	2.1 ± 0.2	5%
12	**15i**	6.4 ± 1.0	10%
13	**15j**	2.7 ± 0.5	7%
14	**PGMI-004A**	13 ± 0.1	38%

* The inhibition ratios against the other three enzymes were measured at a concentration of 5 μM.

**Table 4:** Antiproliferation activities of representative compounds.

Entry	Compounds	Structures	IC_50_ (μM) *
1	**9h**	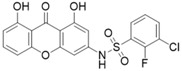	14.1 ± 1.9
2	**9k**	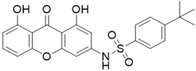	21.1 ± 1.7
3	**9l**	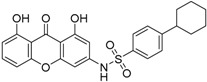	18.5 ± 2.3
4	**9m**	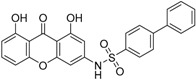	20.1 ± 1.4
5	**15b**	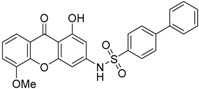	42.7 ± 3.9
6	**15d**	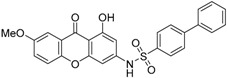	19.6 ± 2.2
7	**15f**	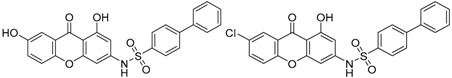	32.7 ± 0.9
8	**15h**	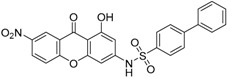	33.8 ± 6.1
9	**15i**		30.6 ± 2.4
10	**15j**	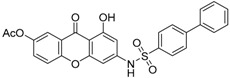	31.1 ± 4.5
Gemcitabine			0.193
PGMI-004A			26.0 ± 2.1

* All IC_50_ values are reported as means ± SD from three determinations.
